# Oncological outcomes of breast cancer patients after planned IORT boost with low-kV x-rays—results of the TARGIT BQR prospective phase IV trial

**DOI:** 10.1007/s00066-025-02412-0

**Published:** 2025-05-19

**Authors:** Lukas Goerdt, Janina Pömsl, Uta Kraus-Tiefenbacher, Viktoria Brück, Christina Kaiser, Ralf Keymer, Yasser Abo-Madyan, Katharina Fleckenstein, Sebastian Berlit, Benjamin Tuschy, Marc Sütterlin, Frederik Wenz, Elena Sperk

**Affiliations:** 1 Department of Gynecology and Obstetrics, University Medical Center Mannheim, Medical Faculty Mannheim, Heidelberg University, Mannheim, Germany; 2 Department of Pediatric and Adolescent Medicine, Medical Faculty, University of Augsburg, Augsburg, Germany; 3 Department of Radiation Oncology, Krankenhaus Nordwest, Frankfurt am Main, Germany; 4 Asklepios Klinik Lich GmbH, Lich, Germany; 5 University Medical Center Bonn, Medical Faculty Bonn, Bonn University, Bonn, Germany; 6 Klinikum Kassel, Kassel, Germany; 7 Department of Radiation Oncology, University Medical Center Mannheim, Medical Faculty Mannheim, Heidelberg University, Mannheim, Germany; 8 Department of Gynecology and Obstetrics, RKH Klinikum Ludwigsburg, Ludwigsburg, Germany; 9 University Hospital Freiburg, Freiburg, Germany; 10 Mannheim Cancer Center, University Medical Center Mannheim, Medical Faculty Mannheim, Heidelberg University, Theodor-Kutzer-Ufer 1–3, Mannheim, Germany

**Keywords:** Intraoperative radiotherapy, Breast neoplasms, Whole-breast irradiation, Breast-conserving surgery, EBRT

## Abstract

**Purpose:**

The TARGIT BQR (boost quality registry) phase IV trial investigates clinical outcomes of breast cancer patients with standard external-beam radiotherapy (EBRT) of the whole breast and intraoperative radiotherapy (IORT) with low-kV x‑rays as an anticipated tumor bed boost in a real-world setting.

**Methods:**

Intraoperative radiotherapy was performed immediately after breast-conserving surgery in one fraction. External-beam radiotherapy and systemic treatment were given according to the German S3 guideline for breast cancer and local tumor board recommendations. Outcome parameters were death, local recurrence, metastasis, local lymph node recurrence, and ipsilateral and contralateral invasive breast cancer. Kaplan–Meier estimates were used to calculate overall survival, metastasis-free survival, local recurrence-free survival, and disease-free survival.

**Results:**

From 10 centers, 1133 patients were recruited. This analysis included 871 patients with 879 cancers, with a median follow-up of 36 months (up to 12 years). An IORT boost was performed in 82% and whole-breast irradiation in 84%. Overall survival was 98.4% after 3 years, 96.8% after 5 years, and 95.4% after 10 years (16 deaths; 1.8%). Metastasis and local recurrence occurred in 11 patients each (1.3%). At 5 years, the local control rate was 97.4% and local recurrence-free survival was 94.4%. Ipsilateral breast cancer occurred in 2 patients, contralateral breast cancer in 3 patients, and local lymph node recurrence in 2 patients. Disease-free survival was 92.9% after 5 years and 82.6% after 10 years.

**Conclusion:**

This phase IV trial confirms previously reported outcomes on upfront IORT boost, with excellent disease-control outcomes.

## Introduction

Breast cancer is still the most common malignant cancer in women worldwide and ranks second in terms of cancer-related deaths after lung cancer [1]. Although global mortality is declining due to better treatment options, the incidence is also increasing due to better screening measures [1, 2].

In general, breast cancer is mostly treated with breast-conserving surgery, which usually involves taking an axillary sentinel lymph node biopsy. In advanced stages, axillary lymph node dissection may also be necessary. In the further course, whole-breast irradiation (WBI) and, if necessary, adjuvant or neoadjuvant systemic therapy is added [3–5]. The risk of recurrence is used as a decisive factor in relation to the choice of treatment in order to divide patients into a high-risk and a low-risk group. Patients with a high risk regarding local relapse receive a boost to the tumor bed in addition to WBI, in accordance with guidelines, including premenopausal patients and postmenopausal patients with risk factors like a tumor size greater than 2 cm, grade 3 tumors, Her2neu-positive tumors, triple-negative tumors, and an extensive intraductal component [6–8]. The importance of boost irradiation in addition to WBI was demonstrated in the EORTC 22881-10882 boost versus no boost trial, which reported improved local control with boost radiation without having an impact on overall survival [9–11]. It was shown that especially young women benefit from the addition of a boost, although a positive effect on local control was seen in all age groups in long follow-up. The most seen side effect was higher-grade fibrosis in the EORTC trial. The boost can be carried out using different techniques and at variable points in time. Boost techniques available include sequential EBRT boost (SEB), simultaneous integrated boost (SIB) during WBI, brachytherapy (as balloon technique or multichannel catheter), and IORT with low-kV x‑rays or electrons during surgery [12–19]. In IORT with low-kV x‑rays (INTRABEAM®, Carl Zeiss Meditec AG, Oberkochen, Germany), a single irradiation of the tumor bed with 20 Gy is carried out immediately after tumor resection. This is followed by WBI with 40–50 Gy, which can be either normo- or hypofractionated [20]. The significant advantages of IORT with low-kV x‑rays compared to most other techniques are that irradiation can begin immediately, there is a minimized risk of geographic miss, and the technique also protects neighboring organs such as the lungs and heart by irradiating a very limited volume of breast tissue around the tumor bed [21–23].

However, or an IORT boost with low-kV x‑rays, no solid prospective data have been published to date for an adequate patient population. Therefore, the prospective TARGIT B(oost) Q(uality) R(egistry) phase IV study (NCT01440010) is the first prospective multicenter study that provides data from a large patient cohort with long follow-up. Here, we show data on oncological outcomes from the TARGIT BQR study.

## Methods

**Fig. 1:**
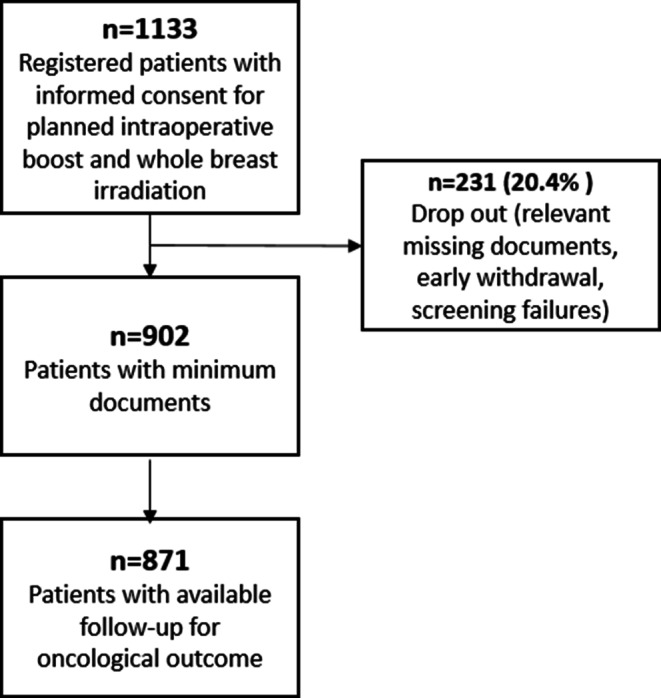
Patient flow in the TARGIT BQR phase IV study and availability of patients for oncological outcome analysis

The TARGIT BQR study included 1133 breast cancer patients from 10 different centers in Germany between September 2011 and December 2020 (Fig. 1). Details regarding recruitment are given in Table 5in the Appendix. The inclusion criteria were broad: patients with breast cancer (cT1 or 2, diameter of the tumor up to 3.5 cm)—irrespective of nodal status, tumor grade, and hormone or HER2 receptor status—and an indication for a tumor bed boost according to the German national S3 guidelines or AGO recommendations before surgery were included. All patients gave their written informed consent to participate in the study. Exclusion criterion was the presence of a multifocal lesion, which usually leads to wider excision cavities. In all patients, the intention was treatment with breast-conserving surgery and IORT as an anticipated boost with a dose of 12 or 20 Gy using low-kV x‑rays (INTRABEAM®) followed by WBI with 40–50 Gy in either normo- or hypofractionation. Indication and treatment implementation were according to local procedures and recommendations of the local tumor board and S3 guidelines. Follow-up was planned 6 months after surgery and then once a year. Outcome variables were local recurrence, ipsilateral breast cancer, contralateral breast cancer, local lymph node metastasis, and distant metastasis. Kaplan–Meier estimates were done to calculate cumulative rates for disease-free survival, local recurrence rate, local recurrence-free survival, metastasis-free survival, and overall survival. For all Kaplan–Meier estimates, death was also counted as an event and not censored, except for the local recurrence rate, where only recurrences were counted as an event. For disease-free survival, any event (death, local recurrence, metastasis, ipsilateral breast carcinoma, contralateral breast carcinoma, local lymph node recurrence) was included, whatever occurred first. Eight patients had bilateral breast cancer and were counted once in case of an event of metastasis or death. Statistical analysis was performed for the endpoints of the oncological outcome in an intention-to-treat setting using SPSS, version 27 (IBM, Armonk, NY, USA). Acute and late toxicity as well as cosmetic outcomes have already been published separately [24, 25].

## Results

### Patient characteristics

A total of 871 patients were included in the current analysis (Fig. 1). Eight patients had bilateral breast cancer and were treated to both sides during the same surgery, resulting in a total of 879 treated cancers. Median age was 61 years, with a range of 30 to 90 years at surgery. Most patients were nonsmokers (median 0 pack years). A detailed description of the characteristics of the whole cohort (*n* = 1133) has been provided previously [24]. Table 1 gives an overview of the characteristics in the current analysis, which did not differ substantially from the whole cohort.

**Table 1:** Postoperative tumor characteristics. Numbers for unknown parameters are not listed separately and represent the rest of patients/treated cancers up to 100%

Tumor characteristics	*N*
*Localization*	
Left breast	444
Right breast	427
*Family history (breast cancer)*	
Positive	236
Negative	636
*Histology*	
No special type (NST)	664
Invasive-lobular	85
Other	52
NST + lobular	4
*T stage*	
0	11
1	605
2	167
3	5
*N stage*	
0	635
1	143
2	17
3	5
*M stage*	
0	747
1	9
*Grade*	
0	5
1	172
2	429
3	184
*L status*	
0	687
1	90
*V status*	
0	725
1	7
*R status*	
0	773
1	20
*Estrogen receptor*	
Positive	710
Negative	91
*Progesterone receptor*	
Positive	681
Negative	117
*HER2neu*	
Positive	84
Negative	720

*T* tumor size according to the TNM classification, *N* involved lymph nodes according to the TNM classification, *M* distant metastasis according to the TNM classification, *Grade* grading according to the TNM classification, *L status* invasion into lymphatic vessels according to the TNM classification, *V status* invasion into veins according to the TNM classification, *R status* resection status according to the TNM classification [26, 27]

### Treatment details

The treatment details of the whole cohort (all recruited patients) have been provided previously [24]. In the current analysis, IORT was given in 717 cases (82%), with a median IORT dose of 20 Gy (10–20 Gy). The applicator size used for IORT was 4 cm (2–5 cm), and the median irradiation time was 24 min (10–55 min). Whole-breast irradiation with EBRT was given in 738 cases (84%) with a median dose of 50.4 Gy (10.8–70 Gy). One patient had no radiotherapy at all, and in 10 cases, no information on IORT and EBRT was provided. For planned but not provided IORT applications, the following reasons were documented: in 21 patients the tumor cavity was too large, in 59 patients the skin distance was too small (< 0.5 cm), in one patient surgery took place in another joint hospital where IORT was not possible (organizational reason), and in 2 patients a technical problem occurred. In the other 71 cases of not performed IORT, no reasons were documented. Overall, 32% of patients received chemotherapy and 80% received endocrine therapy. Treatment details are shown in Table 2.

**Table 2:** Treatment details. Numbers for unknown parameters are not listed separately and represent the rest of patients/treated cancers up to 100%

Treatment	Median/no. patients
**IORT (intraoperative radiotherapy)**	
Dose (median; Gy)	20 (range 10–20)
Irradiation time (median; min)	24 (range 10–55)
Applicator size (median; mm)	40 (range 20–50)
**EBRT (external-beam radiotherapy)**	
Total dose, incl. percutaneous boost if applicable (median; Gy)	50.4 (range 10.8–70)
EBRT single fraction (median; Gy)	1.8 (range 1.6–2.76)
***Chemotherapy (n)***	
Yes	281
No	524
Neoadjuvant	151
***Endocrine therapy regimen (n)***	
*Tamoxifen*	
Yes	302
No	503
*Aromatase inhibitors (AI; n)*	
Yes	166
No	639
*Switch (tamoxifen followed by AI; n)*	
Yes	219
No	586
*Tamoxifen* *+* *GnRH analogue (n)*	
Yes	11
No	794
**Trastuzumab (*n*)**	
Yes	57
No	748

### Oncological outcome

Regarding overall survival in this cohort with a tumor bed boost indication, 96.8% of patients were alive after 5 years and 95.4% after 10 years. There were 16 deaths in total (1.8%). The rate of subsequent metastatic disease was also low, with 11 cases (1.3%) showing distant metastases. This resulted in a metastasis-free survival of 95.8% after 5 years and 88.7% after 10 years. Local recurrences occurred in 1.3% in total (*n* = 11 out of 871 patients) and led to a cumulative local control rate of 97.4% at 5 years and 95.9% at 10 years. Local recurrence-free survival was 94.4% after 5 years and 91.5% at 10 years (27 patients with local recurrence or death as first event). Secondary malignancy in the ipsilateral breast and local lymph node recurrence occurred in 0.2% (*n* = 2) each. Contralateral breast cancer occurred in 0.3% (*n* = 3).

**Fig. 2:**
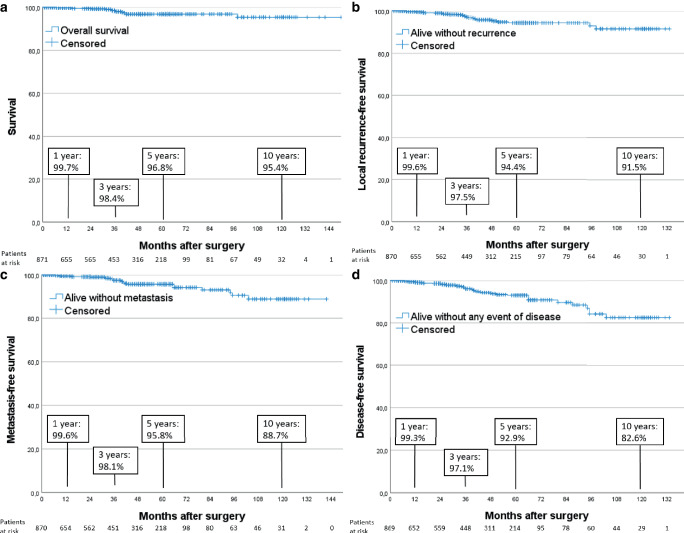
Estimated cumulative rates (Kaplan–Meier curves) for (**a**) overall survival, (**b**) metastasis-free survival, (**c**) local recurrence-free survival, and (**d**) disease-free survival

The Kaplan–Meier curves (Fig. 2) show the estimated cumulative rates of different combinations of events. Due to only few events, no separate cumulative rates were calculated for ipsi- or contralateral breast cancer or local lymph node recurrence. Overall disease-free survival was 92.9% after 5 years in this cohort.

## Discussion

The TARGIT BQR trial is the first multicenter study to provide prospective data on IORT boost using low-kV x‑rays in a large patient cohort. With an overall survival of 98.4% after 3 years and 94.5% after 10 years, a local recurrence rate of 1.3%, and an overall mortality rate of 1.8%, the TARGIT BQR study provides prospective results that consolidate the data already available from retrospective analyses. Remarkable is the low metastasis rate of 11 events throughout the entire follow-up of up to 12 years. The TARGIT BQR study thus shows that IORT with low-kV x‑rays is an effective form of therapy with a very good oncological outcome and is therefore very safe to use.

Table 3 shows a list of the main studies using low-kV x‑rays mainly as an IORT boost. The local recurrence rate at 5 years ranged from 0 to 9.9% percent. The upper range of 9.9% was seen in a cohort with neoadjuvant chemotherapy in high-risk patients [31]. The same group showed an overall survival benefit in patients with neoadjuvant chemotherapy treated with IORT compared to patients without IORT (*p* = 0.028) in a second analysis [35]. Overall survival ranged from 87 to 100% in the studies listed in Table 4. There was one study by Chang et al. showing no local recurrences or deaths in 55 patients after a median follow-up of 40 months [30]. Furthermore, Pez et al. studied larger patient cohorts (*n* = 400) and showed similar ranges and a 5-year LR of 2% with median follow-ups of 78 months and an OS after 5 years of 92.1% [32, 36]. A large meta-analysis by Fan et al., in which a total of 12 studies and over 3000 cases were examined using IORT with low-kV x‑rays as a boost, showed a predicted 5‑year local recurrence rate of 3.45% with a median follow-up of 55 months and, thus, also comparable results [37]. Our prospective data in a large cohort with a low local recurrence rate and high overall survival are in agreement with the available literature.

**Table 3:** Main studies using IORT with low-kV x‑rays as a boost

Author	Year	Follow-up (median; months)	Patients (*n*)	IORT dose at the applicator surface (Gy)	WBI dose (Gy)	Local recurrence rate (%) at 5 years	Overall survival (%) at 5 years
Blank et al. [28]	2010	37	197	20	46–50	2.5	91.3
Wenz et al. [20]	2010	34	154	20	46–50	1.3	87.0
Vaidya et al. [29]	2011	60.5	299	20	45–50	1.73	–
Chang et al. [30]	2014	39.6	55	5 in 1 cm^a^	50	0	100
Kolberg et al. [31]	2017	49	119	20	50	9.9	96.7
Pez et al. [32]	2019	78	400	20	46–50	2	92.1
Sarria et al. [33]	2022	55	653	20	40–50	2.3	91.9
Hochhertz et al. [34]	2023	91.5	68	20	50.4	7	86.8
*Current analysis*	2024	36	871	20	50	97.4	96.8

*WBI* whole-breast irradiation, *IORT *intraoperative radiotherapy

^a^Distance from applicator surface

**Table 4:** Studies using boost techniques other than low-kV x‑rays

Method	Author	Year	Median follow-up (months)	Patients (*n*)	Total dose (Gy; dose per fraction) and technique	Local recurrence (LR; %)/local control (LC; %) follow-up years	Overall survival (%), follow-up years
Brachytherapy	Polgar et al. [38]	2010	133	45	Total dose of 30.3 Gy (*n* = 8) and 36.4 Gy (*n* = 37) in 7 fractions	12-year LR: 9.3%	12-year OS: 88.9%
	Quero et al. [39]	2016	122	621	44 Gy WBI + 5 Gy × 2 fractions HDR BT	10-year LR: 7.4%	10-year OS: 91%
	Guinot JL et al. [40]	2024	62	81	Total dose of 25 Gy (*n* = 33) in 4 fractions and 22.35 Gy (*n* = 48) in 3 fractions	–	5‑year OS: 98.8%
SIB	Hörner-Rieber et al. [41]	2020	61	502	Boost: 28 × 0.5 Gy	2‑year LC: 99.6%	2‑year OS: 99.6%
SEB	Hörner-Rieber et al. [41]	2020	61	502	Boost: 16 Gy in 2 Gy fractions	2‑year LC: 99.6%	2‑year OS: 99.6%
IOERT	Kaiser et al. [18]	2018	121	770	IOERT dose 5–12 Gy WBI dose 54 Gy	10-year LC: 97.2%	10-year OS: 85.7%
	Ciabattoni et al. [42]	2022	57	797	IOERT dose 9–12 Gy WBI dose 50 Gy (75.5%) 40.5/42.56 Gy (23.7%)	5‑year LR: 1.63%	5‑year OS: 98.6%
	Leonardi MC et al. [43]	2024	140	109	IOERT dose 12–21 Gy	5‑year LR: 12.2%	5‑year OS: 95.2%
Percutaneous electron boost	Bartelink et al. [11]	2015	206.4	2661	Percutaneous electron boost 16 Gy WBI dose 50 Gy	20-year LR: 12%	20-year OS: 59.7

*SIB* simultaneous integrated boost, *SEB* sequential external boost, *IOERT* intraoperative electron radiotherapy

Other boost techniques are also in the range of findings from our study, as shown in Table 4. One of the largest and oldest studies (*n* = 2661) in terms of adding a boost to WBI comes from the EORTC, with an LR rate of 12% and an OS of 59.7% after 20 years of follow-up [11]. The higher LR and the lower OS in this study can be explained by the very long follow-up period as well as by differences in adjuvant systemic therapy and must be put into perspective. In a randomized trial (IORT boost with electrons vs. no IORT boost), Ciabattoni et al. showed comparable results, with an in-field and out-field local control of 99.2 and 98.9%, respectively, after 5 years; DFS and OS after 5 years were 96.2 and 98.6%, respectively. Furthermore, in their study, they compared the data from 10 other main studies in the field of intraoperative radiation therapy using electrons (IOERT) [42]. The results for LR ranged from 0 to 11%, and the results for OS ranged from 75 to 99%. Some studies show a certain evidence for IOERT in selected patients [14, 18, 19, 42, 44–48]. The study by Hoerner-Rieber et al. from 2020 compared simultaneous integrated boost (SIB) and sequential boost (SEB) and also showed very good results, with a 2-year local control rate of 99.6% and 2‑year OS of 99.6% for both techniques [41]. Good outcomes are also described in the literature for brachytherapy. Polgar et al. showed a 12-year LR of 9.3% and a 12-year OS of 88.9% in a small sample size of 45 patients with a long median follow-up period of 133 months [38]. The study by Quero et al. from 2016 examined a total of 621 patients and also had a long follow-up period of 122 months. These authors showed a 10-year LR of 7.4% and a 10-year OS of 91% [39].

This study has several limitations that should be considered during interpretation of the data. Firstly, significant advances have been made in systemic therapy in the meantime, particularly with the introduction of new targeted therapies and immunotherapies that may, accordingly, not be represented in our cohort. Furthermore, hypofractionation has now become common practice and could demonstrate different efficacy and side effect profiles in modern treatment approaches compared to those considered in TARGIT BQR. In addition, simultaneous integrated boost regimens may reduce the time-saving aspect of IORT boost in the overall treatment time. Nevertheless, IORT remains the fastest radiation approach delivered immediately after tumor resection.

More robust results are awaited from the randomized TARGIT B trial (NCT01792726) that compares IORT boost with low-kV x‑rays to an external beam boost in a randomized setting [49].

What all techniques and studies have in common, however, is that overall satisfactory oncological results are achieved, and the studies and techniques have all been established as safe procedures. Our analysis adds new prospective data to the field to strengthen shared decision-making, with one of the largest cohorts and a long follow-up.

## Conclusion

This phase IV trial confirms previously reported outcomes on upfront IORT boost, with excellent disease-control outcomes.

## Appendix

**Table 5:** Numbers of recruited patients and treated breast cancers per center and for the whole cohort

Participating centers	Registered patients (*N*)	Patients in current analysis (*N*)	Patients with both-sided breast cancer and treatment in current analysis (*N*)
*All centers*	1133	871	8
Neuss	298	261	3
Mannheim	263	243	2
Frankfurt	180	114	0
Lich	166	92	0
Weinheim	148	112	3
Lüneburg	37	33	0
Osnabrück	28	5	0
Kassel	6	5	0
Bonn	6	5	0
Pinneberg	1	1	0
